# An Account of the Practice of One of the Physicians of the Westminster General Dispensary, and of the Western Dispensary, from the 20th of April, to the 20th of May, 1806

**Published:** 1806-06

**Authors:** Samuel Fothergill

**Affiliations:** Southampton Street, Strand


*An Account of the Practice of one of the Physicians of the Westminster General Dispensary, and of the Western Dispensary, from the 20th of April, to the 20th of May, 1806.*

| | | | |
|---|---|---|---|
| Peripneumony - - | 2 | Insanity - - - | 1 |
| Acute Rheumatism - | 3 | Hypochondriasis - | 1 |
| Hæmoptoe - - | 2 | Palpitation of the Heart | 1 |
| Catarrh - - - | 7 | Asthenia - - | 10 |
| Typhus - - - | 1 | Epilepsy - - - | 2 |
| Synochus - - - | 2 | Ascites - - - | 2 |
| Inflammatory Sore-Throat | 2 | Hydrothorax - - | 1 |
| Tertian - - - | 1 | Gravel and Dysuria - | 4 |
| Hemicranium - - | 1 | Scirrhous Liver - | 1 |
| Pulmonary Consumption | 6 | Enterodynia - - | 2 |
| Cough and Dyspnœa | 28 | Dyspepsia - - | 4 |
| Chronic Rheumatism | 6 | Diarrhœa - - | 3 |
| Lumbago - - | 4 | Hæmorrhoids - - | 1 |
| Sciatica - - - | 2 | Scrophula - - | 3 |
| Cephalalgia - - | 8 | Distorted Spine - | 1 |

Diseased

| | | | |
|---|---|---|---|
| Diseased Vertebræ - | 1 | Amenorrhœa - - | 4 |
| Syphilis - - - | 2 | Leucorrhœa - - | 5 |
| Herpes - - - | 3 | Menorrhagia - - | 1 |
| Scald-head - - | 3 | Hysteria - - - | 1 |
| Itch - - - | 4 | Diseases of Children | 10 |
| Chlorosis - - - | 3 | | |

The diseases which have fallen under my notice during the last month, have generally been mild in their symptoms, and favourable in their progress and termination. Pulmonary and catarrhal affections are still the prevailing complaints; they are, however, slowly yielding to the influence of the fine weather, with which we have lately been favoured; an influence highly propitious to the exertions of the physician.

Several children have been affected with inflammation of the lungs, the measles, and hooping cough. Ophthalmia has not been unfrequent; and some cases of synochus accompanied with diarrhœa, have occurred.

One of the cases of psora, recorded this month, was rather severe, having continued a considerable length of time. The subject of it was a young child, whose parents, not conceiving the eruption to be the itch, paid little attention to it, till they became alarmed on a different account. A medical friend of the family playing with the child, observed the eruption under its clothes, and inquired of the mother, whether it had had the measles lately? No; but it had the cow-pox two years ago, was the reply. The cow-pox! Indeed! And pray, madam, how long after the inoculation might this humour break out? Eighteen months. This was sufficient to alarm the mother: it was impossible the child could have the itch; and no humour had been in the family for some generations. Of course, the complaint was attributed to the cow-pox; and I had some difficulty in dissipating the fears of the mother, and convincing her, much against her inclination, that the itch alone was the occasion of the child's unpleasant appearance and sensations. The only circumstance which caused any doubt as to the nature of the complaint was, that scarcely any eruption appeared between the fingers; and the child had slept with different people, without infecting them.

From this case, we may learn the extreme importance of being guarded in our expressions respecting the cow-pox; the minds of parents have lately been cruelly agitated by the doubts which have been too successfully insinuated uated uated as to its efficacy; and without any hint from the opposers of vaccination, many people are sufficiently ready to attribute every future ailment of their children to the vaccine inoculation. In some instances, the cow-pox has failed from the inoculator not having had a medical education. We cannot sufficiently admire those worthy characters of both sexes, who visit the poor and administer to their wants; some of these have long practised vaccine inoculation. The human body, however, is subject to various cutaneous affections, which are often extremely difficult to distinguish and classify. The chicken-pox sometimes assumes an appearance more similar to small-pox, than to its own character. The vaccine pustules vary in their appearance, and cannot always be depended upon; hence the necessity of experienced practitioners alone being employed. However we may venerate the extensive humanity, and admire the liberal intentions of the *irregular* vaccinators, our regard for the reputation of the cow-pox, and our love for the general welfare of mankind, must induce us to hope that regular practitioners of medicine alone, may be the instruments of diffusing the blessing of vaccine inoculation; whilst we trust that those who are not of the faculty, will content themselves with promoting this most laudable purpose by example, precept, and pecuniary assistance. The cow-pox inoculation is now become a national concern, encouraged by the most illustrious characters, and sanctioned by the majority of medical practitioners; our reputation is implicated in the event, and we should individually give our aid, and use our utmost exertions, to trace all reports of unsuccessful cases to their origin, which will generally be found to lie in error and mistake, or to arise from some improper motive on the part of the author.

SAMUEL FOTHERGILL.

*Southampton Street, Strand,*
*May* 23, 1806.

